# Outbreak of dermatophilosis in horses possibly transmitted by sharing riding equipment

**DOI:** 10.29374/2527-2179.bjvm002124

**Published:** 2024-09-06

**Authors:** Alfredo García Sánchez, Sofia Gabriela Zurita, Maria Gil Molino, Francisco Eugenio Martin Cano, Carmen Barraso Gil, Javier Hermoso de Mendoza Salcedo

**Affiliations:** 1 Veterinarian, Departamento de Producción Animal, Centro de Investigaciones Científicas y Tecnológicas de Extremadura (CICYTEX) Guadajira, Spain.; 2 Veterinarian, Servicio de Procesado de Muestras y Diagnóstico, Hospital Clínico Veterinario, Facultad de Veterinaria, Universidad de Extremadura, Cáceres, Spain.; 3 Veterinarian, Unidad de Reproduccion, Facultad de Veterinaria, Universidad de Extremadura, Cáceres, Spain.; 4 Veterinarian, Red de Grupos de Investigación en Recursos Faunísticos, Instituto de Biotecnología Ganadera y Cinegética (INBIO) Facultad de Veterinaria, Universidad de Extremadura, Cáceres, Spain.

**Keywords:** dermatophilosis, indirect transmission, fomites, equine center, individual stable, dermatofilose, transmissão indireta, fômites, centro equino, estábulo individual

## Abstract

The aim of this study is to describe an outbreak of dermatophilosis at an equestrian center in Castilla la Mancha (central Spain), which affected 16.6% (5/30) of the animals. Research was carried out to establish the mode of transmission and spread to other horses in the herd. Clinical features, diagnostic methods and treatment are also described.

## Resumo

O objetivo deste estudo é descrever um surto de dermatofilose em um centro equestre em Castilla la Mancha (centro da Espanha), no qual 16,6% (5/30) dos animais foram afetados. Foi realizada uma pesquisa para estabelecer o modo de transmissão e a disseminação para outros cavalos do rebanho. As características clínicas, os métodos de diagnóstico e o tratamento também são descritos.

## Introduction

Dermatophilosis is a bacterial cutaneous condition, first described in 1915 by van Saceghem in cattle, which affects a wide variety of domestic and wild mammals, reptiles and humans (van Saceghem, 1915). The disease has a worldwide distribution, an associated with humid environments, and prevails in tropical areas. In has been known in Spain since its first description by Hermoso de Mendoza et al. (1985), who reported outbreaks in horses and sheep in the province of Cordoba (southern Spain).

Dermatophilosis is caused by *Dermatophilus congolensis*, a gram-positive, facultative anaerobic, branching actinomycete (Marsella, 2014) that generally affects only the epidermic tissue and is characterized by purulent exudation, generalised or localized crusts formation and hair loss.

## Case description

**Figure 1:**
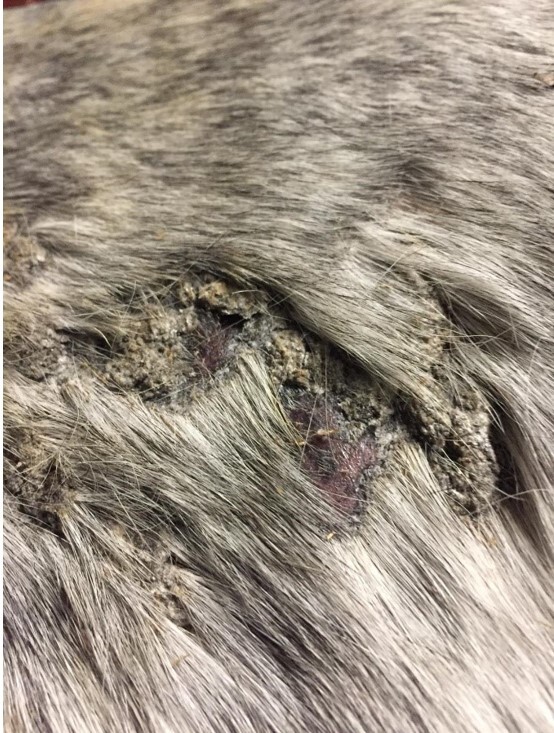
Macroscopic aspect of mare lesion.

The infection was first detected in December 2020, following a period of prolonged rain, and affected a five-year-old Spanish Arabian mare. Lesions were localized on the back and flanks, with the saddle area being the most affected. These lesions included areas of alopecia and thick crusts agglutinating groups of hairs (“paintbrush” lesions) (Figure 1). The removal of scabs was painful and revealed erythematous, ulcerated, or hemorrhagic areas under the crusts, nevertheless, itch signs were not evident. The lesions in the saddle area preventing the mare being ridden.

**Figure 2:**
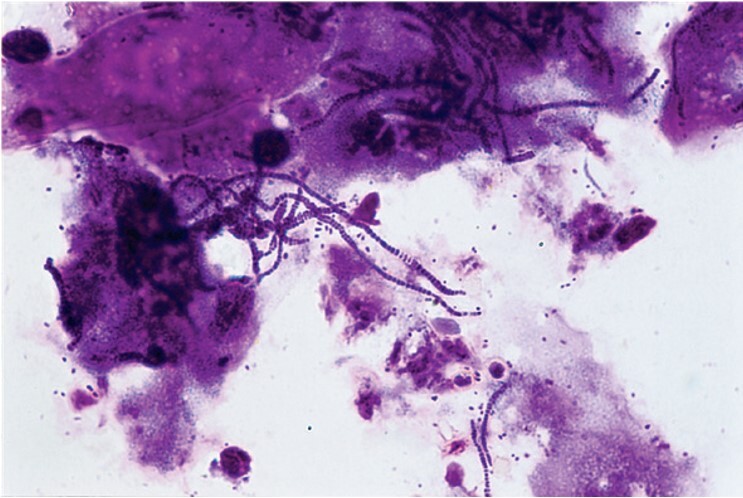
Microscopical examination of Gram stained smears of the scab revealed numerous filamentous forms of *D. congolensis*.

Diagnosis was made based on clinical findings and microscopic examinations of aseptically collected skin scrapings and crusts from the lesion. A Gram-stained smear of the scab material rehydrated in sterile saline, revealed characteristic Gram-positive septate branching filaments, with transversal and longitudinal segmentation (Figure 2).

**Figure 3:**
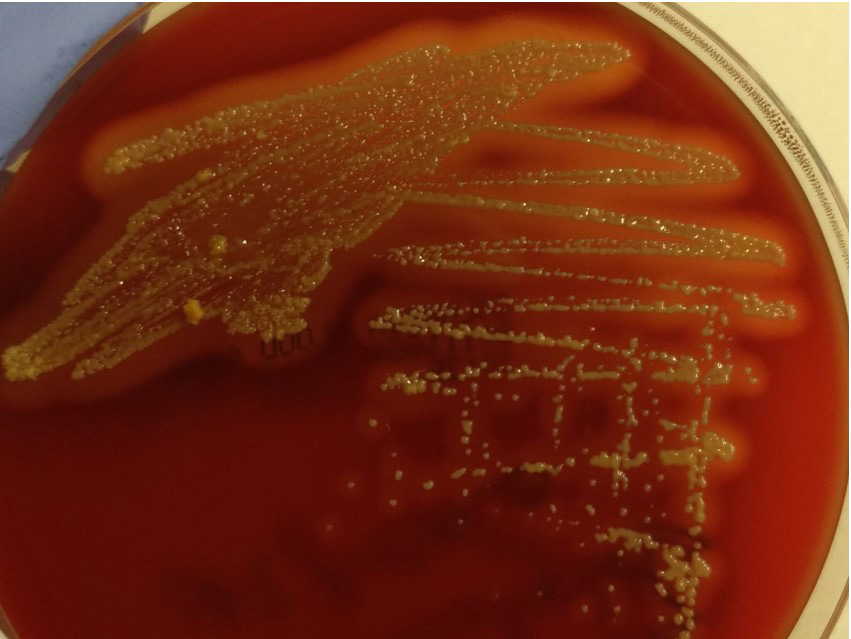
Growth of *Dermatophilus congolensis* in blood agar.

After rehydration in sterile saline, material from the scabs was also inoculated onto sheep blood agar and brain heart infusion agar. The plates were incubated at 37 °C for 48 to 72 hours under 5% CO_2_. Growth of *D. congolensis* in the laboratory is relatively slow (taking 2-3 days), but their distinctive colonies are easy to recognize, as they are often surrounded by an area of beta haemolysis (Figure 3). Although culture plates showed no growth after 24 hours, some round or irregular tiny, creamy yellow-colored, beta-hemolytic colonies adherent to the media grew in blood agar after 48 hours of incubation.

*Dermatophilus congolensis* can live only in the living layers of the epidermis. In comparison to pathogens that reach the dermis, isolation of the organism from the affected skin is relatively simple. However, one of the major problems in isolating *D. congolensis* is avoiding contamination with other bacteria. Its specific isolation using Haalstra’s method with slight modifications (World Organisation for Animal Health, 2008) has been described. However, careful microbiological technique combined with a thorough examination of plates after 48 hours of incubation can also be a good method for successful detection of isolated colonies.

The mare was isolated and treated successfully with a single intramuscular injection of (dihydro) streptomycin/penicillin at a dose of 50,000 U/kg procaine penicillin and 50 mg/kg dihydrostreptomycin combined with topical treatment of lesions with povidone-iodine solution for ten days. A problem with the treatment of bacterial skin infections is that systemic antibacterial therapy (even when the bacterium is known, and its susceptibility is established by an antibiogram), often fails to reach MIC at epidermal level. Even after large doses, treatments often appear to have little effect (Ramey & Baus, 2012).

Over the following few weeks, although the horses were mainly housed in individual box stalls and the mare was specifically isolated from contact with other horses, four more horses showed similar lesions of proliferative and exudative dermatitis with subsequent crusting. The morbidity rate reached 16.6%.

Skin scrapings and scabs were collected aseptically from all horses with active skin lesions on different parts of their bodies and examined for the presence of *D. congolensis*. In all cases, the microscopic examinations of smears also revealed the presence of the actinomycete. Cultures were performed on sheep blood agar plates at 37 °C for 48 hours with 5% CO_2_ (Quinn & Markey, 2003). In two horses, *D. congolensis* was the dominant isolate, but in two other cases, polymicrobial growth was obtained. In fact, it was not possible to isolate *D. congolensis*, which is typically overwhelmed or blocked by the overgrowth of secondary organisms (Outerbridge & Ihrke, 2003). The diagnosis of dermatophilosis was confirmed with a previously described (García et al., 2013) real time-PCR (qPCR) technique, using DNA extracted directly from the skin scabs.

It has been shown that *D. congolensis* is inhibited *in vitro* by the presence of normal skin bacteria, such as *Bacillus* spp. Difficulties in isolating *D. congolensis* explain the scarcity of reports of dermatophilosis cases (Covarrubias et al., 2015).

The primary source of infection is chronically affected animals. When the lesions in these animals became moist, the infective form, the zoospores, are released. Transmission occurs by direct contact with infected animals, although contaminated environments and insects are also suspected to have a role in indirect transmission. Fomites are also an important source of indirect contagions, given that *D. congolensis* in the dry scabs can persist in the environment for long periods (up to 42 months) in favourable conditions (Markey et al., 2013). Several factors are involved in the pathogenesis of dermatophilosis; among which are mechanical injury to the skin, rainfall, or a host with an immune system compromised by tick infestation, concurrent diseases and stress (Ambrose, 1996; Macêdo et al., 2008). The outbreak might have been associated with the exposure of the animals to fomites contaminated with small fragments of infected dry scabs (Marsella, 2014). Fomites such as saddles, bridles and girths, as well as brushes used on the horse's body, can act as vehicles for horse-to-horse transmission, facilitated by small injuries that contact with these items can cause in the epidermis. This seems to be the most probable route in the case described, as direct horse-to-horse contact was rarely observed because the horses were kept in individual stalls. Dry scabs and crusts from affected animals represent an important source of contagion are usually involved in the spreading of lesions on the same animal and possibly the infection of other animals in the same herd.

Molecular techniques using 16S rRNA sequence analysis are increasingly used to identify bacteria to the species level and to diagnose unusual bacterial agents from clinical specimens. Genomic DNA from the three isolates was obtained using the QIAamp DNA Mini extraction kit (Qiagen) according to the manufacturer's instructions. A PCR assay of the *D. congolensis* 16S rRNA gene was performed using the primers previously described by Oladunni et al. (2016) and the products were sent for sequencing. The nucleotide sequence alignment was 100% identical to the 16S rRNA gene of *D. congolensis*, strain: NCTC 13039, sequence ID: LT906453.1.

**Figure 4:**
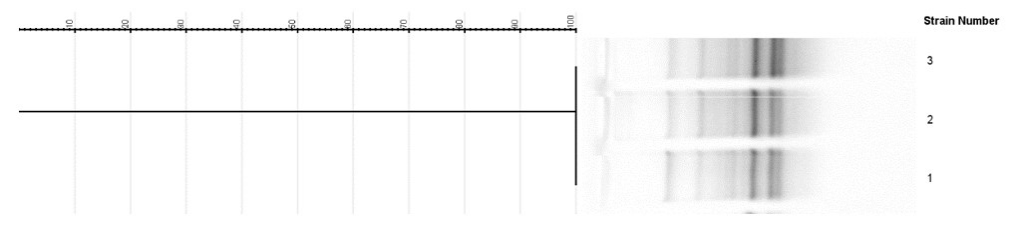
UPGMA dendrogram showing RAPD profiles. All isolates generating a specific profile of DNA fragments.

Random amplification of polymorphic DNA (RAPD) PCR with the primer A (5’-CTTCACCTCGTTGTCCACCC-3’), previously described (García-Sánchez, et al., 2004) was used to analyze the three *D. congolensis* isolates recovered from horses. The RAPD result showed that all the isolates displayed a single RAPD profile (Figure 4). This result establishes the transmission of the strain between horses, and the lack of direct contact between animals reveals the shared use of grooming tools and saddles as the most likely route of transmission and spread of the disease. Sweat under blankets or saddle harnesses and showering after exercise are typical skin moistening situations that can facilitate the spread of zoospores.

**Figure 5:**
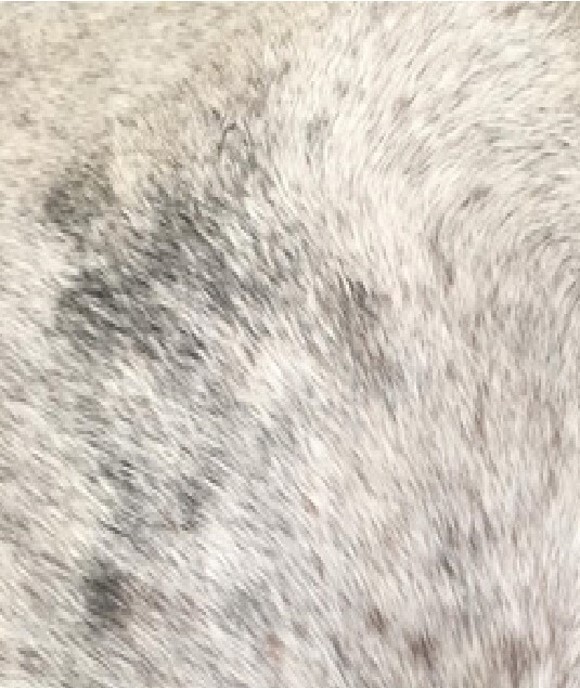
Healing of skin lesions after treatment.

All clinically ill horses were treated with a single intramuscular injection of 50,000 U/kg procaine penicillin and 50 mg/kg dihydrostreptomycin, while the lesions were treated with povidone-iodine solution. Infected horses were carefully cleaned to remove scabs, and individual grooming equipment was used only for infected animals with systematically disinfected after its use (Figure 5).

In horses, crusty lesions should be gently soaked with/in physiological saline solution before removal. A topical antibacterial shampoo is effective as an adjuvant treatment and chlorhexidine-based shampoos are recommended as effective, non-irritating disinfectants (Moriello, 2019). Removal of the scabs allows the skin underneath to receive oxygen, which helps the healing process. The removal of the scabs also promotes hair growth. Furthermore, after their removal, scabs should be destroyed followed by careful cleaning and disinfection of the floor (White, 2005).

## Conclusion

Most horse owners should be aware that a practical way to reduce the risk of contagious equine diseases is to avoid contact with sick horses, but they must also take precautions to avoid the shared use of riding equipment or horse-care tools, such as grooming equipment, saddles, bridles and girths, which have the potential to become contaminated from a horse that is shedding a contagious bacteria or virus. Each animal should have its individual equipment, and if necessary, horse handlers should take the time to clean and disinfect grooming equipment before using it on a new horse. This will help to reduce the potential spread of these organisms.

Finally, it is important to know that *Dermatophilus* can be transmitted to humans and that direct contact with an infected animal can result in infections on hands, arms and even legs.
